# Comparative Evaluation on the Effects of Three Pediatric Syrups on Microhardness, Roughness and Staining of the Primary Teeth Enamel: An In-Vitro Study

**DOI:** 10.7759/cureus.42764

**Published:** 2023-07-31

**Authors:** Divya Mukundan, Vignesh R

**Affiliations:** 1 Pediatric Dentistry, Saveetha Dental College, Saveetha Institute of Medical and Technical Sciences, Chennai, IND

**Keywords:** primary teeth, syrups, staining, roughness, erosion

## Abstract

​​​​​Introduction

One of the most prevalent oral diseases is dental caries. Syrups are commonly used by children who have difficulty swallowing tablets and capsules. Some medications can make dental caries worse and cause the enamel to erode. Most of the time, parents are unaware that a number of foods, drinks, and syrup-formulated pediatric drugs include sugar, which can cause erosion of primary teeth, resulting in plaque accumulation and dental caries. Henceforth, the aim of this study is to assess the effects of pediatric syrups on the microhardness, roughness, and staining ability of primary tooth enamel as a result of daily intake of syrup.

Methods

Eighty primary teeth, including both anterior and posterior, that required extraction due to pre-shedding mobility were randomly divided into four groups of twenty samples each. Samples were exposed to multivitamin syrup (Rudimin), iron syrup (C Pink), and diuretic syrup (Furosemide). The samples were submerged in 10 mL of the respective medication once daily for five minutes for 21 days. On days 0 and 21, enamel surfaces were assessed for changes in microhardness, roughness, and staining. The microhardness was assessed using a Micro Vickers microhardness tester machine; the roughness was assessed using a Mitutoyo surface roughness tester; and the staining ability was assessed using a spectrophotometer, and data processing was done using the analysis of variance (ANOVA) test and Tukey's post hoc analysis.

Results

The changes in enamel surface for microhardness, roughness, and staining were assessed twice, at days 0 and 21. Group 4 (Furoped) showed a significant difference in surface microhardness and staining ability of enamel surfaces with a statistically significant p-value (<0.05). When roughness was compared, all three interventions had a significant difference from the control group, whereas there was no statistically significant difference between the intervention groups.

Conclusion

This study concludes that pediatric syrups can significantly weaken the enamel of primary teeth, resulting in loss of enamel surface microhardness and roughness making them vulnerable to caries. Since frequent consumption of these syrups is correlated with tooth decay, certain precautions like oral hygiene maintenance and rinsing with water after taking the syrup should be done to prevent dental caries, as consumption of medicinal syrups by children cannot be avoided.

## Introduction

One of the most prevalent oral diseases is dental caries. Although there are many theories about how it begins, the one that is generally accepted is that the acid produced by bacteria fermenting carbohydrates, primarily from food, causes dental caries. However, some medications can make dental caries worse and cause the enamel to weaken [1].

Dental erosion, an irreversible breakdown of the enamel's structure without the presence of any microorganisms, can be brought on by both intrinsic and extrinsic sources [1]. Increased intake of acidic food and increased consumption of acidic drinks are some of the primary extrinsic sources of erosion; however, acidic medications have also been one of the causes of dental erosion. Medications can cause tooth erosions through direct contact with dental surfaces or systemic effects. Some drugs, like acidic medications or antihistamines, can weaken tooth enamel, and dry mouth resulting from certain medications reduces saliva's protective effect, leading to tooth erosion [1,2].

Liquid medicines are commonly used by children who have difficulty swallowing tablets and capsules. Liquid medicine usually comes in many forms, which may include solutions, suspensions, and syrups. To act as buffering agents and keep the consistency of syrup, acids are usually added to the syrup, which will also ensure the compatibility of the syrup. Since many syrups have a low pH, which is less than the critical pH, they result in dental erosion [3,4].

Primary and permanent dental enamel have different morphologies, with primary enamel being less mineralized than permanent enamel and having a different structural arrangement [5]. Hence, the primary enamel is more susceptible to erosion when exposed to an acidic environment [6]. Other anatomical structures that play a role in the dissemination of erosion are the tongue, lips, and cheek. In addition to these, saliva has a major role in erosion. Studies have also revealed an association between the count of Streptococcus mutans, the principle microbe responsible for dental caries, and antibiotic use in children between one and three years of age, which is thought to be the most crucial time for acquiring these bacteria.

Several illnesses, such as vitamin deficiency, anemia, and heart ailments, will necessitate daily medication intake, and for children under the age of three, medications are typically administered in the form of syrups. Daily consumption of these syrups can result in dental erosion [4,5]. Most of the time, parents are unaware that a number of syrup-formulated pediatric drugs include sugar. The regular consumption of these syrups can result in enamel degradation [7,8]. There are only a few studies that measure the erosive potential of pediatric syrups, and this study was done to assess the erosion of primary enamel as a result of the daily intake of syrup. The objective of this study is to assess the effects of pediatric syrups on the microhardness, roughness, and staining ability of primary tooth enamel as a result of daily intake of syrup.

## Materials and methods

The current undertaken study is an in vitro study carried out in the Department of Pediatric and Preventive dentistry and approved by the Institutional Review Board of Saveetha University bearing approval no: SRB/SDC/PEDO-2101/22/033. This study compares the erosive potential, i.e., microhardness, roughness, and extrinsic staining, of three commonly used pediatric syrups that are consumed by children under three years at least once a day. The medications included in this study are Rudimin (multivitamin syrup), C pink (iron syrup), and Furoped (Diuretic syrup). The uniqueness of this research is, it examines the effects of diuretic, vitamin, and iron syrups, which are often consumed by children over a prolonged period of time each day. In contrast, earlier research only utilized syrups used for a short time. The purpose of the study is to provide insight into the long-term consequences of these syrups on dental health.

Sample preparation

Eighty primary teeth, including both anterior and posterior, that required extraction due to pre shedding mobility were included in the study. The selected teeth were caries free, without any developmental defects, and non-restored. Scaling was done on the extracted tooth to remove any debris. The tooth specimens were placed in artificial saliva to maintain the natural environment. The samples were randomly allocated into four groups of 20 each. Group 1: Control - distilled water; Group 2: Rudimin - multivitamin syrup (Brio bliss manufacturer); Group 3: C Pink - iron syrup (manufactured by cipla); Group 4: Furoped - Diuretic syrup (manufactured by Samrath life sciences). These medications were chosen because they are frequently used for an extended period of time in pediatric patients.

Baseline Assessment

**Figure 1:**
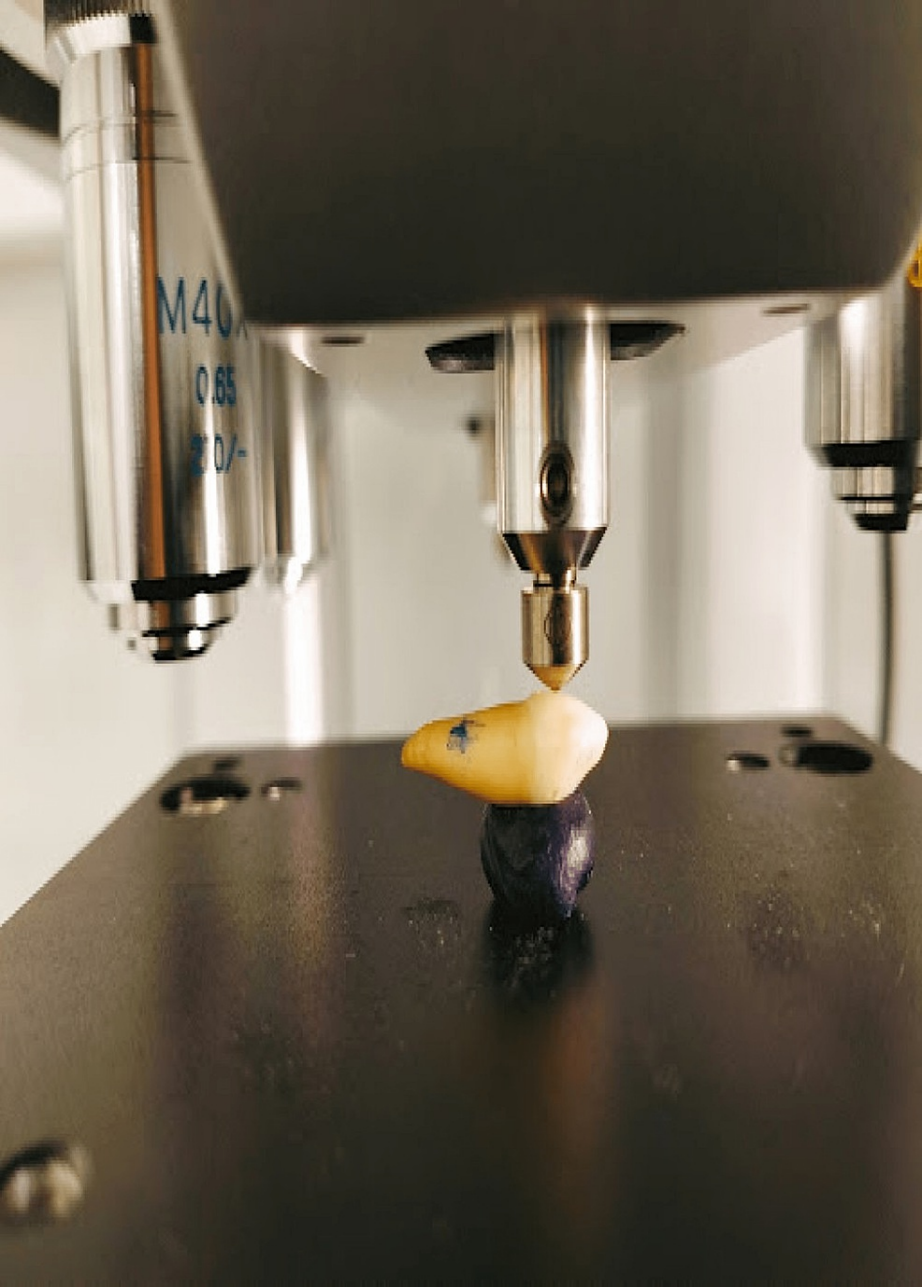
Microhardness being measured on the buccal surface of the specimen's flattest area using Micro Vickers microhardness tester machine.

Before the immersion cycle, the initial microhardness of the tooth specimen was assessed using the Micro Vickers microhardness tester machine. It consists of a diamond indenter, and a 25g force was applied to the specimen's flattest area on the buccal surface to measure microhardness as seen in Figure 1.

**Figure 2:**
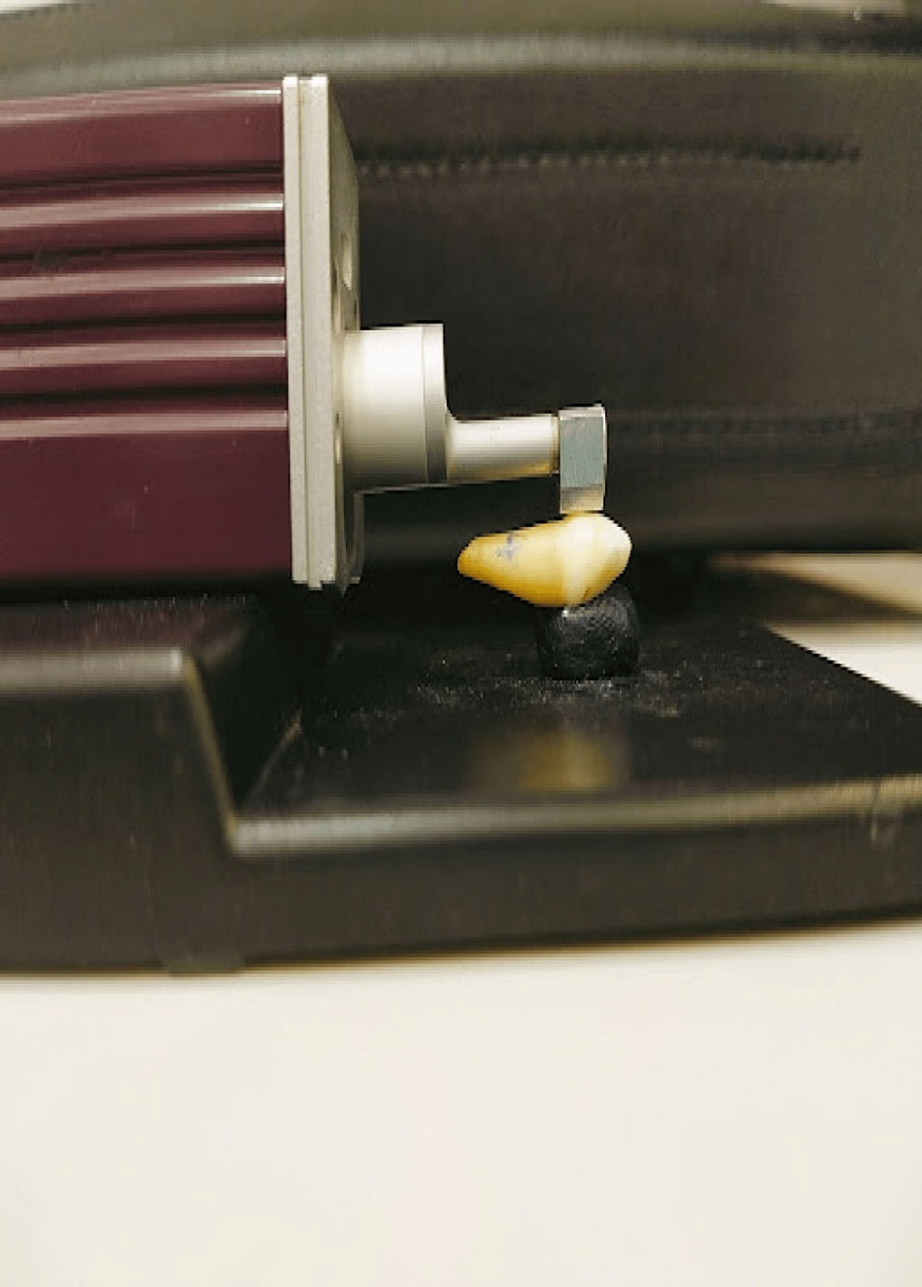
Roughness was measured on the buccal surface of the specimen's flattest area using Mitutoyo surface roughness tester.

Roughness (Ra value) was assessed using a Mitutoyo surface roughness tester. Roughness was measured on the buccal surface of the specimen's flattest area as seen in Figure 2.

**Figure 3:**
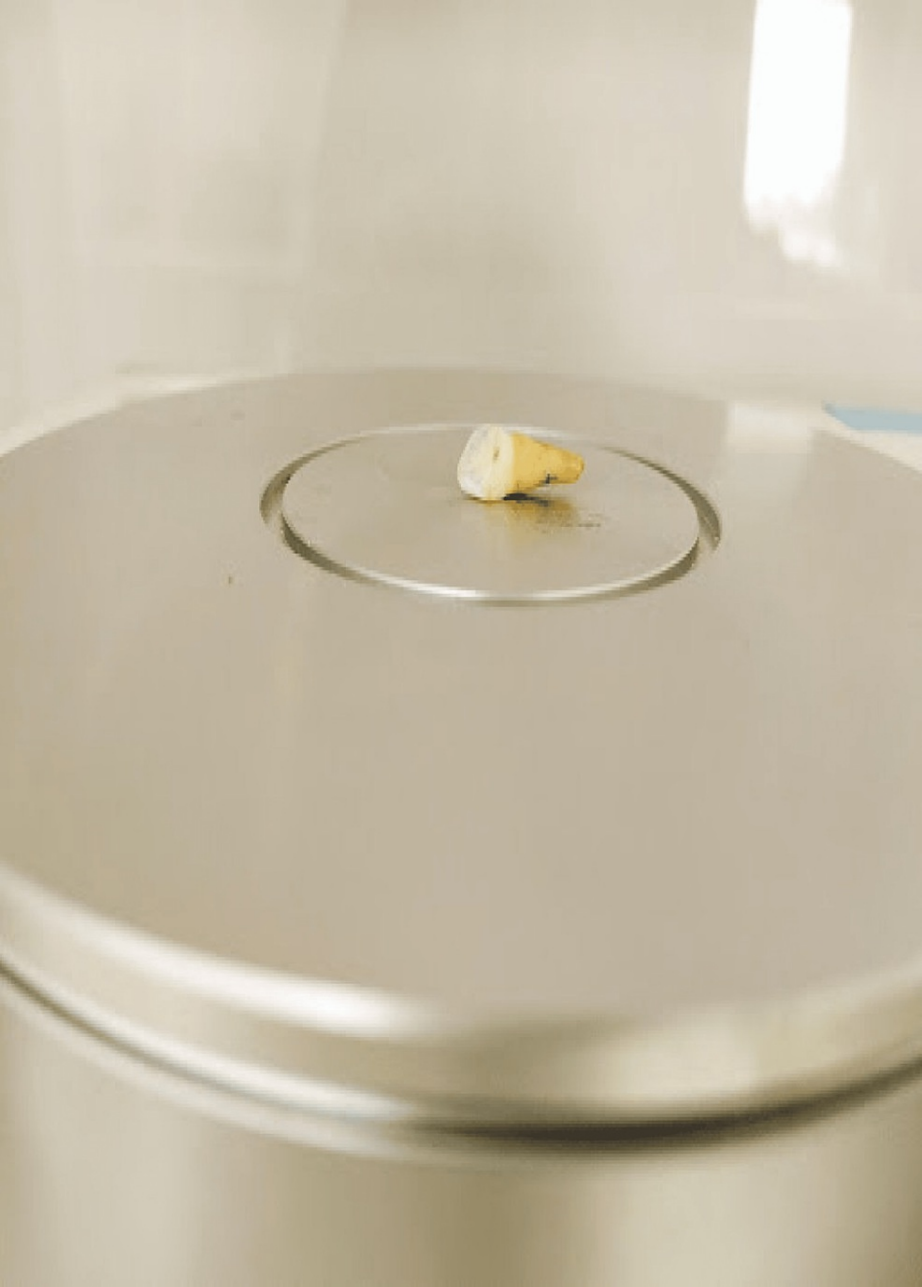
The shade of tooth specimens being measured using a spectrophotometer on the exposed buccal surface.

The baseline shade of tooth specimens was measured using a spectrophotometer. The shade was measured on the exposed buccal surface as seen in Figure 3.

Specimens were submerged in 10 ml of the respective medication once daily for 21 days, with the exposed area exposed for one minute. The specimens were cleaned with distilled water after each cycle of immersion and placed in artificial saliva until the next immersion. 

Statistical analysis

The surface microhardness, roughness, and staining of the specimens were analyzed and tabulated using SPSS software (IBM Corp., Armonk, NY). The means of the experimental and control groups were compared using one-way ANOVA, and then Tukey's post hoc analysis was used to identify differences between the groups. A p-value ≤0.05 was considered statistically significant.

## Results

All three liquid medications showed a gradual change in surface microhardness, roughness, and staining over a period of three weeks when compared to the control group. When post-op enamel surface microhardness for all groups was observed, Group 4 (Furoped) has the highest post-immersion loss of microhardness, and the least was shown by iron syrup. A statistically significant difference was found in group 4, compared to the other three groups (Table 1).

**Table 1:** Comparison of enamel surface microhardness post-op using Tukey's post hoc test

(I) Groups	(J) Groups	Mean Difference (I-J)	Sig.
Control	Vitamin drops	-37.60000^*^	.095
	Iron drops	-17.70000	.091
	Diuretics	73.90000^*^	.000
Vitamin drops	Control	37.60000^*^	.095
	Iron drops	19.90000^*^	.045
	Diuretics	111.50000^*^	.000
Iron drops	Control	17.70000	.091
	Vitamin drops	-19.90000^*^	.045
	Diuretics	91.60000^*^	.000
Diuretics	Control	-73.90000^*^	.000
	Vitamin drops	-111.50000^*^	.000
	Iron drops	-91.60000^*^	.000

When post-op enamel surface roughness for all groups were observed, all three interventions had a significant difference from the control group, whereas there was no statistically significant difference between the intervention groups (Table 2).

**Table 2:** Comparison of enamel surface roughness post op using Tukey's post hoc test

(I) Groups	(J) Groups	Mean Difference (I-J)	Sig.
Control	Vitamin drops	-.435100^*^	.000
	Iron drops	-.496400^*^	.000
	Diuretics	-.456450^*^	.000
Vitamin drops	Control	.435100^*^	.000
	Iron drops	-.061300	.742
	Diuretics	-.021350	.985
Iron drops	Control	.496400^*^	.000
	Vitamin drops	.061300	.742
	Diuretics	.039950	.912
Diuretics	Control	.456450^*^	.000
	Vitamin drops	.021350	.985
	Iron drops	-.039950	.912

When post-op enamel surface staining for all groups were observed, Group 4 (Furoped) had the highest post-immersion staining. A statistically significant difference was found in group 4, compared to the other three groups (Table 3).

**Table 3:** Comparison of enamel surface staining post op using Tukey's post hoc test

(I) Groups	(J) Groups	Mean Difference (I-J)	Sig.
Control	Vitamin drops	4.93000	.147
	Iron drops	3.18000	.511
	Diuretics	12.77500^*^	.000
Vitamin drops	Control	-4.93000	.147
	Iron drops	-1.75000	.871
	Diuretics	7.84500^*^	.005
Iron drops	Control	-3.18000	.511
	Vitamin drops	1.75000	.871
	Diuretics	9.59500^*^	.000
Diuretics	Control	-12.77500^*^	.000
	Vitamin drops	-7.84500^*^	.005
	Iron drops	-9.59500^*^	.000

## Discussion

Dental erosion is the chemical loss of tooth surface hard tissue by acids as a result of external factors like food sources and medicines, as well as intrinsic factors like gastric reflux [1,2]. These days, dental erosion is considered a significant factor in tooth structure loss in both adults and children. One of the main reasons for erosion in children is the frequent intake of syrups. Acids are typically added to the syrup to act as buffering agents and maintain the consistency of the syrup. Dental erosion happens as a result of the low pH of many syrups, which is below the threshold pH [1,3,6]. Despite citric acid being a weak acid, it is a powerful erosive agent and the principal acid that is most frequently employed in syrups. Children who have trouble swallowing tablets and capsules frequently take liquid medications. A liquid drug typically comes in a variety of forms, including syrup, suspension, and solutions. Several studies have suggested that medications consumed as syrups can change the tooth's morphological pattern [8,9]. Sucrose is the most commonly used sweetener in syrup because it is easily processed. Other sweetening agents, such as glucose and fructose, are also added. In comparison to glucose and fructose, sucrose plays a significant role in erosion because it serves as a substrate for the oral bacteria that cause fermentation, produce acids, and lower the intraoral pH [10]. A few studies have concluded that there is a correlation between syrup and erosion of enamel, which can result in dental caries [11,12]. Apart from morphological changes in the enamel, a few syrups can also cause extrinsic staining of the teeth, which is a concern to parents and will also affect the child's social interaction. Various factors like viscosity, frequency of intake, and bedtime intake of syrup also play a key role in dental erosion [5]. Taking these medications right before bed can erode the teeth since the salivary flow will be lowered during sleep, which will restrict the saliva's ability to clean the teeth. Hence, the aim of this study is to assess the effects of pediatric syrups on the microhardness, roughness, and staining ability of primary tooth enamel as a result of daily intake of syrup.

Studies done by Chandran et al., Scatena et al., and Namkar et al. showed that pediatric liquid medications can cause some change in the morphological pattern of enamel, such as loss of microhardness [3,8,13]. In the present study, microhardness, surface roughness, and staining were evaluated as changes in surface morphology may contribute to bacterial colonization of teeth and plaque development. Hardness was assessed using the Micro Vickers hardness tester machine, roughness (Ra value) was assessed using the Mitutoyo surface roughness tester, and the baseline shade of all tooth specimens was measured using a spectrophotometer. Both anterior and posterior teeth were included and analyzed, and assessments were made on the buccal surface of all teeth.

In this study, Furosemide syrup (Diuretic) presented the highest erosive and staining potential, showing a substantial increase in microhardness and staining when compared to Rudimin (vitamin syrup) and C Pink (iron syrup). When surface roughness was compared, all groups showed statistically significant differences when compared to the control group.

According to Valinoti et al.'s study of the erosive effects of various acidic drugs, deciduous tooth enamel microhardness decreased, which is in accordance with the findings of this study [14]. Deciduous teeth are reported to be more susceptible to an acidic and cariogenic environment than permanent teeth, mostly due to variations in the thickness, mineralization, and structural makeup of the enamel. The enamel and dentin are less mineralized when compared to permanent teeth, and primary teeth have a larger dental pulp chamber, which could lead to rapid caries [4,15]. The results of this study show that liquid pediatric medicines can contribute to enamel disintegration. Further long in vivo studies are required to establish better results, as in in-vitro research, oral conditions cannot be precisely stimulated.

There are a few limitations in this study such as, the sample size is relatively small, and the study does not include other classes of pediatric syrups, resulting in an insufficient evaluation of the erosive effects across all categories of the drugs. Future research should consider using a larger and more diverse sample size, comprising a wider variety of pediatric syrups.

## Conclusions

This research is unique in that it investigates the effects of vitamin, iron, and diuretic syrups on the erosion of primary enamel as a result of daily syrup intake over a longer period of time than previous studies, which only investigated syrups taken for a shorter period of time. This study concludes that pediatric syrups can significantly weaken the enamel, as there is a loss in microhardness and an increase in roughness, making them vulnerable to caries. Since frequent consumption of these syrups is correlated with tooth decay, certain precautions should be taken to prevent dental caries, as the consumption of medicinal syrups by children cannot be avoided.

To minimize the risk of tooth decay, it is recommended to rinse the mouth with water after taking the syrup. This helps to remove any residual sugar or acidity from the teeth and minimize the potential damage to the enamel. Additionally, maintaining good oral hygiene is crucial. Regular brushing and flossing, along with routine dental checkups can help maintain healthy teeth and prevent caries. By implementing these precautions, parents and caregivers can prevent the negative effects of pediatric syrup on children's teeth and promote better oral health.
